# Selective Serotonin Reuptake Inhibitor Antidepressant Treatment Discontinuation Syndrome: A Review of the Clinical Evidence and the Possible Mechanisms Involved

**DOI:** 10.3389/fphar.2013.00045

**Published:** 2013-04-16

**Authors:** Thibault Renoir

**Affiliations:** ^1^Florey Institute of Neuroscience and Mental Health, Melbourne Brain Centre, University of MelbourneMelbourne, VIC, Australia

**Keywords:** antidepressant treatment, selective serotonin reuptake inhibitor, SSRI discontinuation syndrome, SSRI withdrawal syndrome, animal models, clinical evidence, serotonin

## Abstract

Besides demonstrated efficacy, selective serotonin reuptake inhibitors (SSRIs) hold other advantages over earlier antidepressants such as greater tolerability and a wider range of clinical applications. However, there is a growing body of clinical evidence which suggests that SSRIs could, in some cases, be associated with a withdrawal reaction upon cessation of regular use. In addition to sensory and gastrointestinal-related symptoms, the somatic symptoms of the SSRI discontinuation syndrome include dizziness, lethargy, and sleep disturbances. Psychological symptoms have also been documented, usually developing within 1–7 days following SSRI discontinuation. The characteristics of the discontinuation syndrome have been linked to the half-life of a given SSRI, with a greater number of reports emerging from paroxetine compared to other SSRIs. However, many aspects of the neurobiology of the SSRI discontinuation syndrome (or SSRI withdrawal syndrome) remain unresolved. Following a comprehensive overview of the clinical evidence, we will discuss the underlying pathophysiology of the SSRI discontinuation syndrome and comment on the use of animal models to better understand this condition.

## Introduction

Selective serotonin reuptake inhibitors (SSRIs) are widely used in the treatment of depressive disorders (Petersen et al., 2002; Chaudhry et al., 2011) and anxiety disorders (van der Linden et al., 2000; Hedges et al., 2007). Total SSRI prescription volume increased threefold between 1995/1996 and 2006/2007 (Lockhart and Guthrie, 2011). Despite this, the use of SSRIs is not without flaws. Apart from potential adverse side effects arising from long-term antidepressant treatment, as well as issues of relapse, and recurrence following remission of a depressive episode (Rucci et al., 2011; Rush et al., 2012), the existence of a SSRI discontinuation syndrome (also known as SSRI withdrawal syndrome) has been suggested in some cases. A decade ago, Harvey et al. (2003) highlighted that although significant progress had been made to uncover the pathophysiology of depression and the mechanisms of actions of SSRIs, the neurobiology of SSRI discontinuation syndrome had not been comprehensively addressed. Following an overview of the clinical evidence, we will discuss the possible molecular mechanisms implicated in the pathology of SSRI discontinuation syndrome, and comment on the use of animal models to better understand this condition.

## Clinical Evidence of SSRI Discontinuation Syndrome

About 15 years ago, Zajecka et al. (1997) proposed a definition of the SSRI discontinuation syndrome as the onset of a cluster of symptoms following the discontinuation of a SSRI, not attributable to other causes (i.e., concomitant medication, illness). Along with sensory and gastrointestinal symptoms, the SSRI discontinuation syndrome includes somatic symptoms such as dizziness, lethargy, and sleep disturbances, as well as psychological symptoms such as anxiety/agitation, irritability, and poor concentration (Warner et al., 2006; Haddad and Anderson, 2007). The literature on these symptoms initially consisted mainly of case reports (Coupland et al., 1996; Price et al., 1996; Schatzberg et al., 1997; Bryois et al., 1998; Goldstein et al., 1999). Haddad (1997) reviewed 47 separate reports of SSRI discontinuation syndrome, 30 of which involved paroxetine compared to only seven involving fluoxetine, despite the latter being prescribed more frequently. Black et al. (2000) identified 53 different symptoms within the condition (with dizziness being the most common) and proposed a diagnostic criteria for SSRI discontinuation syndrome that requires two or more of the described symptoms developing within 1–7 days of discontinuation (or reduction in dosage) of an SSRI after at least 1 month of treatment.

These case reports have since been followed up by a number of controlled studies (i.e., prospective studies, with a randomized, double-blind interruption period, which included a systematic method for discontinuation symptoms data collection). For example, using the Discontinuation Emergent Signs and Symptoms (DESS) checklist, Rosenbaum et al. (1998) assessed the effects of a 1-week placebo substitution period in patients diagnosed with unipolar depressive disorder who had been maintained on fluoxetine, sertraline, or paroxetine for similar periods of time (∼11 months). Following treatment interruption, there was a significant increase in the number of DESS items observed in the sertraline- and paroxetine-treated patients. In contrast, this was not detected in fluoxetine-treated patients. Another double-blind trial found that placebo substitution for paroxetine was associated with increased frequency and severity of specific physical and psychological symptoms, which arose as early as after the second missed dose (Michelson et al., 2000). In that trial, patients with a history of depression had been undergoing SSRI treatment for similar periods of time (∼12–15 months) and were equivalent on several parameters (e.g., similar baseline symptom severity). The discontinuation symptoms seemed to be specific to paroxetine in this study, since patients treated with sertraline or fluoxetine did not exhibit discontinuation syndrome within the 5-day placebo substitution period. However, since sertraline and fluoxetine (but not paroxetine) have active metabolites with half-lives around 2–3 and 7–15 days respectively (Table 1), the effects of longer withdrawal periods are worth assessing in future studies. In the case of fluoxetine, it is possible that discontinuation symptoms may emerge when the levels of the active metabolite decrease after 15 days and beyond. Consistent with that possibility, Zajecka et al. (1998) found a small increase in reports of dizziness among patients who discontinued fluoxetine 4 and 6 weeks after placebo substitution. Bogetto et al. (2002) investigated 97 outpatients diagnosed with dysthymic disorder who were instructed to stop their medication (paroxetine or fluoxetine) after a successful treatment period of at least 8 weeks. With a mean time of onset of symptoms of 2 days after drug discontinuation, discontinuation syndrome was reported by 27% of patients (of which 85% had been treated with paroxetine compared to only 15% treated with fluoxetine). Analyzing randomized controlled studies of escitalopram and paroxetine for the treatment of anxiety disorders (in which treatments were followed by a prospectively defined discontinuation period), Baldwin et al. (2007) reported that individuals taking either SSRI showed more discontinuation symptoms compared to placebo. Following a 12–24 week treatment period, patients were abruptly switched to placebo and were assessed using the DESS checklist 1 and 2 weeks after drug cessation. Overall, paroxetine withdrawal induced significantly more discontinuation symptoms than escitalopram.

**Table 1:** **Pharmacokinetic and pharmacological parameters of SSRIs (adapted from Hiemke and Hartter, 2000; Owens et al., 1997, 2001; Tatsumi et al., 1997)**.

Compounds	Half-life (mean)	SERT (human)	5-HT_1A_ (rat)	5-HT_2A_ (rat)	5-HT_2C_ (porcine)	NET (human)	DAT (human)	Muscarinic (human)
Fluvoxamine	15 h	*K*_D_: 2.2, *K*_i_:2.3	n.d.	n.d.	*K*_i_:5786	*K*_D_: 1300, *K*_i_:1427	*K*_D_: 9200, *K*_i_:16790	*K*_i_:31200
Paroxetine	20 h	*K*_D_: 0.13, *K*_i_:0.10	*K*_i_:21168	*K*_i_:6320	*K*_i_:9034	*K*_D_: 40, *K*_i_:45	*K*_D_: 490, *K*_i_:268	*K*_i_:72
Sertraline	26 h	*K*_D_: 0.29, *K*_i_:0.26	*K*_i_:3663	*K*_i_:2207	*K*_i_:2298	*K*_D_: 420, *K*_i_:714	*K*_D_: 25, *K*_i_:22	*K*_i_:427
Citalopram	36 h	*K*_D_: 1.16, *K*_i_:1.6	n.d.	n.d.	*K*_i_:2051	*K*_D_: 4070, *K*_i_:6190	*K*_D_: 28100, *K*_i_:16540	*K*_i_:1430
Fluoxetine	1–4 days	*K*_D_: 0.81, *K*_i_:1.1	*K*_i_:8313	*K*_i_:141	*K*_i_:72	*K*_D_: 240, *K*_i_:599	*K*_D_: 3600, *K*_i_:3764	*K*_i_:702
Desmethylsertraline	2–3 days	*K*_D_: 3.0	n.d.	n.d.	n.d.	*K*_D_: 390	*K*_D_: 129	n.d.
Norfluoxetine	7–15 days	*K*_D_: 1.47	n.d.	n.d.	n.d.	*K*_D_: 1426	*K*_D_: 420	n.d.

*Equilibrium dissociation (*K*_D_) and inhibition (*K*_i_) constants for binding at serotonin, norepinephrine and dopamine transporters (SERT, NET, and DAT, respectively) and several selected neurotransmitter receptors were determined using radioligand binding assays. n.d.: not determined*.

There are a variety of symptoms associated with paroxetine withdrawal as Murata et al. (2010) recently provided based on observations of a group of Japanese outpatients (*n* = 56). These symptoms included dizziness (50% of patients), increased dreaming/vivid dreams (35%), fatigue (30%), nausea/vomiting (30%), headache (25%), anxiety/agitation (25%), paresthesia (15%), insomnia (10%), diarrhea (10%), visual disturbances (10%), fever (10%), tremor (5%), irritability (5%), and chills (5%). In addition to these symptoms, in an assessment of 87 patients who had their treatment interrupted for 4–7 days in a double-blind placebo study, Hindmarch et al. (2000) also reported greater cognitive deficits, poorer quality of sleep and increased depressive symptoms associated with paroxetine discontinuation, symptoms which were not evident in patients ceasing fluoxetine, sertraline, or citalopram treatment.

To date, there is no evidence to suggest that the length of SSRI treatment is associated with the development of more symptoms or with the severity of those symptoms (Rosenbaum et al., 1998; Michelson et al., 2000; Baldwin et al., 2007). However, several studies suggest that an abrupt interruption of treatment results in more symptoms of the discontinuation syndrome compared to a gradual tapering of the drug (van Geffen et al., 2005; Himei and Okamura, 2006; Murata et al., 2010). This suggests that the incidence, timing, and severity of SSRI discontinuation symptoms may be related to plasma elimination characteristics of each drug. In view of this, Michelson et al. (2000) found a statistically significant relationship between the percentage reduction in plasma concentration of the drug and the appearance of discontinuation symptoms resulting from placebo substitution across all drug groups (fluoxetine, sertraline, and paroxetine). Comparing drug concentrations before and after placebo substitution, Henry et al. (2000) found that lower steady-state brain levels of paroxetine (but not fluoxetine) after placebo substitution were associated with greater risk for discontinuation-related adverse events. Overall, discontinuation symptoms are more frequent after the abrupt cessation of drugs with shorter half-lives (Rosenbaum et al., 1998; Bogetto et al., 2002; Judge et al., 2002; Baldwin et al., 2007). However, that relationship may not be an absolute predictor. Indeed, despite paroxetine having a similar half-life to that of fluvoxamine, the rate of withdrawal reactions for the latter drug is 10 times lower (Price et al., 1996). For example, in the 46 patients with discontinuation symptoms reviewed by Black et al. (2000), paroxetine was stopped in 65% of these cases compared to only 7% of cases with fluvoxamine. To date, there are no double-blind trials with placebo substitution comparing the effects of discontinuation from paroxetine and fluvoxamine, and the higher number of reports of withdrawal reactions with paroxetine is likely to reflect the greater prescription rate of paroxetine over fluvoxamine (Lockhart and Guthrie, 2011). Other factors (i.e., pharmacological) could also contribute to the high frequency of discontinuation syndrome with paroxetine.

Notably, paroxetine has significant off-target effects, including an affinity for cholinergic receptors similar to the tricyclic antidepressants (Owens et al., 1997, 2001), raising the possibility for a cholinergic rebound during discontinuation. Fujishiro et al. (2002) further demonstrated that the ability of paroxetine to induce an anticholinergic effect in mice was similar to that of tricyclic antidepressants, whereas fluvoxamine was much less potent in that regard. Paroxetine also exhibits moderately high affinity for the norepinephrine transporter, unlike other SSRIs (Tatsumi et al., 1997). In comparison, the longer half-lives of the active compounds of fluoxetine and sertraline may be the reason underlying fewer symptoms of the discontinuation syndrome. However, as raised previously, the major limitation of most of the clinical studies examining the discontinuation syndrome is the restriction of treatment interruption to only few days, when longer periods (i.e., weeks) might be more suitable for the study of fluoxetine withdrawal. Finally, whether or not the pharmacodynamic characteristics of a drug could also help to explain the clinical observations remains to be addressed. For instance, fluoxetine is also a 5-HT_2C_ receptor antagonist (Sanchez and Hyttel, 1999) with affinity for 5-HT_2A/2C_ receptors (see Table 1), whereas sertraline is the sole SSRI which also inhibits the dopamine transporter (Tatsumi et al., 1997).

The discontinuation syndrome has been reported in relation to nearly every SSRI, although there are increased reports of occurrence in patients ceasing treatment of paroxetine. However, the full extent of discontinuation syndrome might only emerge if future studies are designed to take into account the pharmacokinetics of the drug and availability of its active metabolite. Double-blind trials with placebo substitution have yet to be undertaken to directly compare the effects of discontinuation from SSRIs with shorter half-lives (i.e., paroxetine and fluvoxamine). Overall, the crucial question of whether the discontinuation syndrome is equally related to the lack of specificity and/or the neurobiology related to the half-life of the SSRI remains unclear. Potential genetic-drug interactions should also be systematically assessed in future studies. Indeed, a recent clinical study suggested a possible involvement of the C(-1019)G polymorphism of the serotonin 5-HT_1A_ receptor gene in the occurrence of paroxetine discontinuation syndrome, as patients with the −1019C allele experienced paroxetine discontinuation syndrome more frequently than patients who were −1019G homozygous (Murata et al., 2010).

## Animal Studies

### Mechanisms of action of SSRIs

Numerous laboratories have studied the effects of SSRIs on the serotonergic system, mainly investigating on the pharmacological characteristics of these drugs (Table 1). For example, using intracerabral microdialysis in conscious rodents, extracellular concentrations of serotonin have been reported to be increased following acute administration of fluoxetine (Hervas and Artigas, 1998), citalopram (Rea et al., 2010), or sertraline (Kitaichi et al., 2010). Further, Sharp et al. (1997) showed that paroxetine induced a larger increase in extracellular serotonin in the frontal cortex when serotonin autoreceptors on both the somatodendrites (5-HT_1A_) and nerve terminals (5-HT_1B_) were blocked. Incidentally, the acute effects of SSRIs on serotonin neurotransmission are controlled by several feedback mechanisms, which include 5-HT_1A_ and 5-HT_1B/1D_ autoreceptors (Malagie et al., 2001; Pullar et al., 2004) as well as postsynaptic 5-HT_2_ receptors (Boothman et al., 2003; Cremers et al., 2004; Calcagno et al., 2009). Recent discoveries suggest an unexpected complexity in the mechanisms controlling the activity of serotonin neurons (Sharp et al., 2007).

Notably, and particularly relevant to this review, antidepressant drugs typically require chronic administration (i.e., several weeks) to achieve therapeutic efficacy. This delay is thought to partly reflect the time required for autoreceptors to desensitize so as to facilitate serotonin neurotransmission (Kreiss and Lucki, 1995). Rather than the desensitization *per se*, Le Poul et al. (1995) suggested that the progressive increase in the number of serotonergic neurons with desensitized 5-HT_1A_ autoreceptors may play a critical role in the slow development of the antidepressant actions of SSRIs. Extensive research has been done to study the changes to serotonergic signaling induced by chronic SSRI administration (summarized in Table 2).

**Table 2:** **Summary of the main serotonergic adaptive changes during chronic administration versus discontinuation from SSRIs (in animal models)**.

Drug treatment	Effects of chronic treatment	Effects of treatment cessation	Reference
Fluoxetine (30 mg/kg/day for 3 weeks)	↓5-HT and 5-HIAA levels in HC and FC	7 day washout: ↓5-HT and 5-HIAA 14 day washout: 5-HT back to normal but ↓5-HIAA levels	Trouvin et al. (1993)
Citalopram for 2 weeks (minipump: 50 mg/ml)	no effect on serotonin turnover (↔5-HIAA/5-HT)	↑5-HIAA/5-HT ratio after a 48-h washout period	Bosker et al. (2010)
Fluoxetine (70 μmol/kg/day for 2 weeks)	↓5-HT and 5-HIAA levels in HC and FC	5-HT and 5-HIAA levels still reduced after 7 day washout	Caccia et al. (1993)
Paroxetine (40 μmol/kg) and Sertraline (40 μmol/kg) for 2 weeks	↓5-HT and 5-HIAA levels in HC and FC	5-HT and 5-HIAA levels back to normal 7 day washout	Caccia et al. (1993)
Fluoxetine (6.9 mg/kg/day for 3 weeks)	↑5-HIAA/5-HT ratio	5-HIAA/5-HT still increased after 8 day washout	Stenfors and Ross (2002)
Paroxetine (5-10 mg/kg/day) and Sertraline (7.5 mg/kg/day)	↓SERT binding after 21 day		Benmansour et al. (1999)
Sertraline (7.5 mg/kg/day)	↓SERT binding after 2–3 weeks of treatment	SERT binding back to basal levels after 10 days of washout	Benmansour et al. (2002)
Citalopram	↓SERT binding after long- term treatment	SERT binding back to basal levels after 48 h withdrawal	Horschitz et al. (2001)
Fluoxetine (3 mg/kg/day)	↓ SERT mRNA in raphe after 7 days of treatment	SERT mRNA in raphe back to control levels after 7 days washout	Neumaier et al. (1996)
Citalopram (for 3 weeks)	↑postsynaptic 5-HT_1A_ binding		Gunther et al. (2008), Klimek et al. (1994)
Fluoxetine (10 mg/kg/day for 2 weeks)	↓8-OH-DPAT-induced oxytocin, ACTH, and CORT responses	The oxytocin response was still reduced 60 days after discontinuation of fluoxetine	Van de Kar et al. (2002), Raap et al. (1999)
Fluoxetine (5-10 mg/kg for 2 weeks)	↓8-OH-DPAT-induced reduction in [5-HT]ext		Dawson et al. (2002), Newman et al. (2004)
Citalopram (20 mg/kg/day) and Escitalopram (10mg/kg/day)	↓8-OH-DPAT-induced reduction in [5-HT]ext after 13 day of treatment		Ceglia et al. (2004)
Fluoxetine (minipump: 1 mg/kg/day for 4 weeks)	↓WAY100635-induced increase in [5-HT]ext	5-HT_1A_ autoreceptor desensitization was sustained after a 2-week washout period	Popa et al. (2010)
Fluoxetine (3 mg/kg/day for 7 day)	↓5-HT_1B_ mRNA in raphe but ↑5-HT_1B_ mRNA in FC and HP	5-HT_1B_ mRNA in raphe back to control levels after 7 day washout	Neumaier et al. (1996)
Paroxetine and Fluoxetine (5 mg/kg/day)	↓5-HT_1B_ mRNA in raphe after 8 weeks of treatment	5-HT_1B_ mRNA back to control levels after 3-14 days of washout	Anthony et al. (2000)
Fluoxetine (5 mg/kg/day for 12 day)	↓CP-93129-induced reduction in [5-HT]ext		Newman et al. (2004)
Citalopram (minipump: 50 mg/ml for 2 weeks)	No change in 5-HT_1B_ sensitivity		Jongsma et al. (2005)
Citalopram (for 3 weeks)	↓Forebrain 5-HT_2A_ binding		Gunther et al. (2008), Klimek et al. (1994)
Paroxetine (10 mg/kg/day) and Fluvoxamine (90 mg/kg/day)	↓mCPP-induced CORT response after 21 days		Yamauchi et al. (2006)
Fluoxetine	↑5-HT_2_ receptor binding in FC and HC		Hrdina and Vu (1993), Klimek et al. (1994)
Fluoxetine (10 mg/kg/day for 21 day)	↑DOI-induced ACTH and CORT response; ↓DOI-induced oxytocin		Damjanoska et al. (2003)
Fluoxetine (10 mg/kg for 21 day)	↑DOI-induced c-fos gene expression in FC and HC		Tilakaratne et al. (1995)
Fluoxetine (for 3 weeks)	↓5-HT_2C_ mRNA in FC↑5-HT_2C_ mRNA in HC	↓5-HT_2C_ mRNA in FC persisted after 1 week withdrawal 5-HT_2C_ mRNA in HC back to basal levels after withdrawal	Barbon et al. (2011)
Fluoxetine (18 mg/kg/day for 24 day) via drin *K*_i_ ng water)	↑5-HT_2C_ pre-mRNA editing in BALB/c (but not C57BL/6) mice		Englander et al. (2005)
Paroxetine (10 mg/kg/day) and Fluvoxamine (90 mg/kg/day)	↓mCPP-induced hypolocomotion		Yamauchi et al. (2004)
Paroxetine and Fluoxetine (10 mg/kg/day p.o. for 21 day)	↓mCPP-induced hypolocomotion		Kennett et al. (1994)

*↑Significant increase; ↓significant decrease; SERT: serotonin transporter; CORT: corticosterone; 8-OH-DPAT: 8-hydroxy-2-(di-*N*-propylamino) tetralin (5-HT_1A_ receptor agonist); WAY100635: *N*-[2-[4-(2-methoxyphenyl)-1-piperazinyl]ethyl]-*N*-2-pyridinylcyclohexanecarboxamide maleate salt (5-HT_1A_ receptor antagonist); DOI: 2,5-dimethoxy-4-iodoamphetamine (5-HT_2A_ receptor agonist); mCPP: meta-chlorophenylpiperazine; CP-93129: 5-HT_1B_ receptor agonist; FC: frontal cortex; HC: hippocampus; [5-HT]ext: extracellular serotonin levels (measured by microdialysis); 5-HIAA: 5-hydroxyindoleacetic acid (the main metabolite of serotonin)*.

Although it was initially thought that the antidepressant effects of SSRIs were solely attributable to an increase in brain serotonin, more recent evidence suggests that a sustained increase in serotonin levels (at least within the hippocampus) does not appear to be required for the beneficial anxiolytic/antidepressant-like effects of chronic fluoxetine (Popa et al., 2010). Studies have now shown that SSRIs modulate other neurochemical signaling systems in the brain such as noradrenaline and dopamine. For instance, paroxetine was reported to increase extracellular levels of noradrenaline in the hippocampus of rats that had received repeated administration of the drug (Hajos-Korcsok et al., 2000). Similarly, chronic (but not acute) treatment with fluoxetine potentiates dopamine D2/D3 receptor function (Collu et al., 1997; Dziedzicka-Wasylewska et al., 2002). Therefore, it has now become evident that in addition to effects at receptors regulating monoamine release (e.g., 5-HT_1_, 5-HT_2_, and α-2 adrenergic receptors), multiple targets (e.g., glutamate/GABA, peptidergic systems, neurotrophic factors, hypothalamic-pituitary-adrenal axis, etc.) are likely to be involved in the mechanism of action of SSRIs (Schechter et al., 2005; Renoir et al., 2012). With that in mind, we next review the effects of SSRI treatment discontinuation in animal models. Most of these reports have focused on the serotonergic system. However, as mentioned, there is scope for future studies to assess non-serotonin signaling pathways in relation to the SSRI discontinuation syndrome.

### Effects of SSRI treatment discontinuation in animal models

In this section we first focus on the few animal studies which report behavioral changes after cessation of SSRI treatment (versus behavioral changes found during treatment), followed by a discussion of the potential molecular mechanisms involved. Despite the relatively consistent cluster of clinical symptoms observed in the SSRI discontinuation syndrome, few animal studies have been conducted to explicitly examine the effects of withdrawal from chronic SSRI treatment. Moreover, most of those studies have used fluoxetine, despite a low incidence of discontinuation syndrome associated with this specific SSRI in the clinic.

In one animal study, contrasting with the reduction of locomotor activity observed during chronic administration of fluoxetine (30 mg/kg/day for 5 days), fluoxetine-treated rats showed a significant increase in locomotor activity upon discontinuation (Bjork et al., 1998). Notably, this “rebound effect” was only observed during the first 4 h following the first “missed” dose, and not during the subsequent washout period. These findings suggest that upon cessation of chronic fluoxetine, rats showed an increase in activity resembling withdrawal behavior, in line with the symptoms of anxiety/agitation reported by some patients who develop the discontinuation syndrome. Hyperactivity in response to abrupt fluoxetine discontinuation may result from fluoxetine’s effects on the dopaminergic system, specifically in the ventral tegmental area and in the nucleus accumbens. In that regard, Gardier et al. (1994) found that chronic fluoxetine treatment caused a persistent decrease in striatal dopamine levels lasting up to 14 days after treatment discontinuation. Other reports have provided further evidence of changes in dopaminergic signaling during SSRI discontinuation. By measuring the spontaneous firing rate of hippocampal CA1 neurons, Bijak and Smialowski (1988) found that the excitatory reaction evoked by the selective D1 receptor agonist SKF-38393 was potentiated following repeated citalopram administration, and was further increased after drug withdrawal. Similarly, the enhancement of dopaminergic synaptic modulation (through hippocampal D1 receptor upregulation) induced by chronic fluoxetine was maintained for 1 month after drug withdrawal (Kobayashi et al., 2012). The mesolimbic dopamine system is thought to be involved in the behavioral effects of antidepressants (Collu et al., 1997), as well as in the withdrawal syndrome following cessation of drugs of abuse (Hu, 2007; D’Souza and Markou, 2010; Radke et al., 2011). However, another animal study found no change in intracranial self-stimulation threshold during withdrawal from chronic fluoxetine treatment (Lin et al., 1999). This finding suggests that there is no change in central reward function after cessation of fluoxetine treatment, and therefore that SSRI discontinuation syndrome and withdrawal symptoms in drug addiction may involve distinct pathways. However, since several clinical observations point to the existence of tolerance phenomena during antidepressant treatment in some cases (Fava and Offidani, 2011), further animal research is required to determine the dependence potential of the various types of antidepressant drugs.

In an attempt to compare and contrast the mechanisms underlying the effects of continuous SSRI administration against abrupt SSRI discontinuation, Bosker and colleagues measured the acoustic startle response of rats that had either been receiving 2 weeks of citalopram or had undergone 48 h of discontinuation from citalopram treatment (Bosker et al., 2010). Behavioral assessments started 2 days after the removal of osmotic mini pumps filled with either saline or 50 mg/ml citalopram. Habituation was significantly diminished in the 48-h discontinuation group, as their acoustic startle response (a transient motor response to an unexpected, intensive stimulus) was significantly greater compared to the rats receiving ongoing 2 week citalopram treatment. Exaggerated acoustic startle response has been previously linked to anxiety-like behaviors in rodents (Plappert and Pilz, 2002). Therefore, the findings of Bosker et al. (2010) suggest a higher level of anxiety in the citalopram discontinuation group, which is similar to the increased incidence of anxiety in patients with the SSRI discontinuation syndrome. An increased reactivity to acoustic stimuli in rats has been linked to long-term depletion of serotonin in the brain (Tanke et al., 2008). Consistent with that notion, Bosker et al. (2010) found that an index of serotonin turnover [the ratio of 5-hydroxyindoleacetic acid to serotonin (5-HIAA/5-HT)] was increased after the 48-h washout period following chronic citalopram treatment, while chronic treatment exerted no significant effect on serotonin turnover. This finding is in agreement with an earlier study reporting that levels of the serotonin metabolite 5-HIAA are increased during withdrawal from chronic fluoxetine (30 mg/kg/days for 21 days), and are sustained at levels exceeding control levels (by 30–50%) for at least 14 days after cessation of chronic fluoxetine treatment (Trouvin et al., 1993). Similarly, while serotonin turnover rates were significantly decreased during the first 24 h after the last injection of a 3-week treatment with fluoxetine (6.9 mg/kg/day), turnover rates increased significantly after an 8-day washout period (Stenfors and Ross, 2002). Behavioral changes in relation to increased serotonin turnover rates during drug washout were not examined. Similarly, as Bosker et al. (2010) assessed the biochemical consequences and behavioral effects of citalopram discontinuation in separate cohorts of animals, no direct comparisons between increased serotonin turnover and increased acoustic startle response could be made. Further behavioral testing is required to validate the finding of a greater acoustic startle response, using additional tests such as the elevated-plus maze, the light-dark box, etc. Finally, based on the low incidence of the discontinuation syndrome reported in patients treated with citalopram or fluoxetine, it is likely that studying the role of serotonin turnover in the behavioral effects of long-term paroxetine administration versus discontinuation might give different results. Indeed, likely resulting from the accumulation of its main active metabolite norfluoxetine after 14 days of chronic administration, only fluoxetine (but not paroxetine)-treated animals exhibited reduced brain serotonin levels after a 1-week washout period (Caccia et al., 1993).

Despite the fact that adaptive changes in 5-HT_1A/1B_ autoreceptors are critically involved in the mechanistic actions of SSRIs (as discussed in See Mechanisms of Action of SSRIs), many preclinical studies of SSRI withdrawal have focused on serotonin levels. As such, a lack of understanding of any possible adaptive functional changes to serotonin receptors remains. Raap et al. (1999) reported that 5-HT_1A_ receptors located in the hypothalamus of rats are desensitized following a 2-week treatment with fluoxetine, an effect which lasted for at least 60 days. Given the implications of the fronto-cortical regions and the hippocampus in the withdrawal symptoms, and based on altered serotonin metabolism mentioned in the previous section, further work is required to improve our understanding of the extent of 5-HT_1A_ receptor adaptation in the brain following SSRI withdrawal. One study suggested that molecular changes follow a rapid time course, requiring 3–14 days of washout to reverse the reductive effects of chronic fluoxetine and paroxetine treatment on pre-synaptic 5-HT_1B_ receptor mRNA levels in rat dorsal raphe nucleus (Anthony et al., 2000).

Although not the main focus of the studies, two separate groups reported the behavioral effects of long-term administration of paroxetine compared to the behavioral effects of discontinuation of paroxetine treatment (Gervasoni et al., 2002; Elizalde et al., 2008). It was found that the anxiolytic-like effects of chronic paroxetine (10 mg/kg/day for 3 weeks) were no longer observed after a 2-week washout period (Elizalde et al., 2008). The loss of this drug effect is interesting considering the development of symptoms of anxiety in patients with the discontinuation syndrome. On the other hand, the antidepressant effect of paroxetine and the amelioration of cognitive deficits were maintained even after a 2-week washout period, which does not correspond with increased incidence of depressive symptoms in individuals experiencing the discontinuation syndrome. While this study was not designed to explicitly examine the discontinuation syndrome, to our knowledge this is the sole preclinical study assessing the longer term behavioral effects of halting chronic SSRI treatment in an animal model of depression (i.e., chronic mild stress paradigm). As the eventual development of the discontinuation syndrome was not the focus of the study, other behavioral parameters relevant to the clinical symptoms of the syndrome, such as changes in sleep patterns/architecture, were not examined. Gervasoni et al. (2002) reported that in rats, a reduction in the amount of REM sleep, as well as an increase in the duration of REM sleep episodes, was associated with chronic treatment with paroxetine (5 mg/kg/day for 21 days), rather than to discontinuation of treatment, as these sleep changes were not observed after a 24-h washout period. Since sleep disturbances are often reported in patients with discontinuation symptoms, it is of importance for future animal studies examining the discontinuation syndrome to assess sleep patterns/architecture as relevant outcomes.

Overall, the number of preclinical studies looking at the effects of SSRI after a washout period remains very limited. Many such reports were published prior to the recognition of SSRI discontinuation syndrome as a clinical condition, and were mainly intended to study the mechanism of action of the SSRI while having the drug on board. To date, clear discontinuation-like behaviors in animal models have not yet been reported. However, it should be noted that we suggest that a flaw of these previous studies did not use animal models of depression. In addition, it is of importance that future studies consider an appropriate time point for assessment based on the half-life of the drug of interest. It is also of importance for future studies to examine the effects of paroxetine, as this SSRI has been implicated in numerous clinical cases of the discontinuation syndrome (Bogetto et al., 2002; Judge et al., 2002; Baldwin et al., 2007). The animal studies performed to date have focused on measures of tissue serotonin levels; as it is now clear that other pathways are involved in the mechanistic actions of SSRIs, future investigations should be broadened to include an examination of these additional actions. The complex nature of SSRI discontinuation syndrome, in terms of the variety of subtle symptoms that may present at the clinic, poses a challenge for preclinical studies, and there is a need for appropriate animal models to be developed in order to facilitate further study of the behavioral aspects of this condition. As mentioned, the majority of studies to date have been based on “normal” animals, whereas the clinical population who develop the discontinuation syndrome are typically taking SSRIs for treatment of depression, anxiety, etc. As such, future studies of the SSRI discontinuation syndrome in rodents should encompass well-established animal models of depression, chronic SSRI treatment, followed by an appropriate period of drug withdrawal.

## Discussion and Conclusion

Although initial observations relied only on case reports, there is now a substantial body of clinical evidence suggesting that, in some cases, SSRIs may be associated with a withdrawal response when halted after a period of regular use. The existence of these symptoms, known as the SSRI discontinuation syndrome, has now been confirmed by a number of well-controlled studies (i.e., double-blind randomized placebo-controlled design, in which treatment is followed by a prospectively defined discontinuation period). These studies suggest that the best route of action for cessation of SSRI treatment is to taper down the dose of the medication rather than abrupt termination, as tapering is likely to decrease the possibility of the occurrence of discontinuation symptoms. Due to the broad range of symptoms which can develop as part of the discontinuation syndrome, prior to drug initiation, patients and caregivers need to be provided with adequate education and realistic, objective appraisals of possible outcomes which can develop during and following antidepressant treatment.

The current evidence suggests that the discontinuation syndrome is dependent on the SSRI half-life, with more reports of symptoms occurring in patients treated with paroxetine compared to other SSRIs. However, studies designed to assess the onset of discontinuation syndrome, and how the syndrome coincides temporally with pharmacokinetic withdrawal, are still lacking. Such studies are critical in order to draw conclusions when comparing SSRIs with short versus long half-lives. It is currently unclear as to whether the discontinuation syndrome is equally related to the pharmacological properties of a given SSRI, and/or its half-life. Future studies could also examine possible associations of the syndrome with clinical characteristics, as one study reported that the discontinuation syndrome was more common in patients with earlier onset of dysthymic disorder, and was also more common in females (Bogetto et al., 2002). On the other hand, Baldwin et al. (2007) reported no difference in discontinuation symptoms in patients with depression compared to patients with anxiety disorders. Future clinical investigations need to have enough statistical power to enable examination of within-group comparisons. Current understanding of the pathophysiology associated with the SSRI discontinuation syndrome remains largely speculative (Blier and Tremblay, 2006; Delgado, 2006). In fact, the sole clinical investigation looking at the possible chemical and molecular mechanisms underlying the SSRI discontinuation syndrome was based on a single subject (Kaufman et al., 2003).

Notably, not all patients treated with SSRIs (which represents a very heterogeneous population) experience discontinuation symptoms. In that regard, a recent clinical study indicated a possible involvement of the C(-1019)G polymorphism of the serotonin 5-HT_1A_ receptor gene in the occurrence of paroxetine discontinuation syndrome (Murata et al., 2010). Whether the development of discontinuation symptoms has a genetic component requires additional studies to provide more conclusive results. The use of genetic animal models might be able to shed light in that regard. Interestingly, using mice with higher (1A-High) or lower (1A-Low) autoreceptor levels, Richardson-Jones et al. (2010) found a negative relationship between 5-HT_1A_ autoreceptor level and response to antidepressants. Further studies looking at the effects of 5-HT_1A_ receptor function on vulnerability to the discontinuation syndrome are of interest. Most of the animal studies performed thus far have naturally focused on the serotonergic system, and have mainly used fluoxetine, despite the fact that this specific SSRI has been associated with a lower incidence of discontinuation syndrome in the clinic. However, based on the numerous targets known to be changed adaptively during chronic treatment with SSRIs in animal models, further studies assessing non-serotonergic pathways would be worthwhile. Finally, preclinical studies using appropriate animal models of anxiety/depression are still lacking when it comes to the study of SSRI discontinuation.

## Conflict of Interest Statement

The authors declare that the research was conducted in the absence of any commercial or financial relationships that could be construed as a potential conflict of interest.
